# Professional psychological qualities of Chinese medical students: theoretical models, questionnaire development, and relationship with mental health

**DOI:** 10.3389/fpsyg.2024.1411085

**Published:** 2024-07-04

**Authors:** Wenping Luo, Wenshu Fan, Yanglin Xia, Yanchun Dou, Juan Du

**Affiliations:** ^1^School of Clinical Medicine/Affiliated Hospital, Chengdu University of Traditional Chinese Medicine, Chengdu, China; ^2^Mental Health Education Center for College Students, Chengdu University of Traditional Chinese Medicine, Chengdu, China; ^3^College of Health and Rehabilitation, Chengdu University of Traditional Chinese Medicine, Chengdu, China; ^4^Mianyang Maternal and Child Health Hospital, Mianyang, China

**Keywords:** professional psychological qualities, medical students, questionnaire development, mental health, masking effect

## Abstract

**Introduction:**

Professional psychological qualities are crucial for individuals’ career development and overall well-being, especially in clinical medical professions. Medical students often face significant work, academic, and doctor-patient communication pressures, which can challenge their mental and emotional health. Measuring and understanding the relationship between medical students’ professional psychological qualities and their mental health is of significant practical importance.

**Methods:**

This study developed a comprehensive professional psychological qualities scale through a series of qualitative and quantitative studies, consisting of three main components and thirteen secondary dimensions. The scale’s reliability was assessed using Cronbach’s α coefficients. In Study 2, the scale was administered to 972 medical students to explore their anxiety and depression levels. A simple mediation analysis was conducted to investigate the relationship between professional psychological qualities, anxiety, and depression.

**Results:**

The professional psychological qualities scale demonstrated satisfactory reliability, with a total scale α coefficient of 0.947 and subscale α coefficients ranging from 0.895 to 0.933. The mediation analysis revealed that medical students’ professional psychological qualities directly negatively impact depression levels and indirectly positively influence them via their effects on anxiety levels, exhibiting an overall masking effect unrelated to depression levels.

**Discussion:**

This study addresses the gap in research on the professional psychological qualities of medical students by providing a reliable measurement tool. The findings shed light on the complex mechanisms through which these qualities impact the mental health process. The scale can be used by other researchers to assess medical students’ professional psychological qualities and further investigate their relationship with mental health outcomes.

## 1 Introduction

Since the onset of the COVID-19 pandemic in 2020, healthcare professionals have faced unprecedented challenges and pressures (Saeki and Shimato, 2021). These challenges extend beyond the immediate health threats posed by the virus to encompass issues such as strained healthcare resources, changing work environments, and the anxiety of patients’ families. In the face of regular emergencies in healthcare, medical students are required to make rapid decisions and provide high-quality services. Cultivating professional psychological qualities are crucial in maintaining composure, adjusting strategies, and maximizing the safeguarding of patient health (Zhang et al., 2013). Furthermore, compared to students in other disciplines, medical students often endure intense academic demands, necessitating the maintenance of a positive attitude and efficient learning (Frajerman et al., 2019). In clinical medical practice, medical students may encounter various stressors, including the life-and-death challenges of patient care, urgent tasks, and communication difficulties with patients’ families. These professional characteristics have prompted reflection among educational institutions and scholars regarding talent development in the healthcare industry, highlighting the critical importance of fostering positive professional psychological traits such as emotional stability, strong coping abilities, teamwork, and resilience in medical students (Lin et al., 2019; Ferguson et al., 2022).

Fostering professional psychological quality in medical students is crucial for their well-being and future success as healthcare professionals. Strategies to enhance professional psychological quality may include incorporating stress management and resilience training into medical curricula (Dyrbye et al., 2021), providing mentorship and support programs (Wasson et al., 2016), and promoting a culture of self-care and help-seeking (Ayala et al., 2018). Additionally, incorporating mindfulness-based interventions has shown promise in reducing stress and improving well-being among medical students (Daya and Hearn, 2018). By prioritizing the development of professional psychological quality, medical schools can better prepare students to navigate the challenges of their educational journey and future careers.

The literature extensively explores the correlation between medical students’ professional psychological quality and anxiety. Meta-analyses and studies across different semesters and cultures highlight the prevalence of anxiety and its dynamic fluctuations throughout medical education (Mirza et al., 2021). Factors such as resilience, coping mechanisms, perceived competence, and cultural influences shape anxiety levels (Pereira et al., 2015; Puthran et al., 2016).

Similarly, the relationship between professional psychological quality and depression among medical students has received significant attention. Studies emphasize the high risk of depression and suicidal ideation in this population (Rotenstein et al., 2016). State–trait anxiety mediates the relationship between sleep quality and depression symptoms (Chen et al., 2022), while stress, anxiety, and professional identity are negatively correlated. Medical students experience higher levels of psychological and education-related stress, leading to increased depression and anxiety (Saxena et al., 2019). Poor sleep quality, low self-esteem, and the COVID-19 pandemic further exacerbate these mental health challenges (Kalyani et al., 2017; Chang et al., 2021). Understanding these relationships is crucial for developing interventions and support systems to promote medical students’ mental well-being throughout their training (Slavin, 2016; Tan et al., 2020).

Research on the professional psychological quality of medical students is essential, but it is hindered by the lack of dedicated measurement scales (Wang et al., 2022). While existing research offers valuable insights into the role of resilience in the psychological well-being of medical students, further exploration and the creation of dedicated measurement scales are needed to thoroughly evaluate the professional psychological quality of this population. The present study aims to address this gap by developing a comprehensive scale to measure the professional psychological qualities of medical students and examining its relationship with anxiety and depression levels. By doing so, this research seeks to contribute to a better understanding of the factors influencing the mental health of medical students and inform the development of targeted interventions to support their well-being throughout their educational journey.

Based on the literature review, it is evident that professional psychological qualities play a crucial role in the occupational development and mental well-being of medical students. However, there is a lack of comprehensive measures specifically designed to assess these qualities in medical student populations. Moreover, the complex mechanisms through which professional psychological qualities influence mental health outcomes, such as anxiety and depression, remain underexplored. To address these gaps, the present study aims to develop and validate a scale for measuring professional psychological qualities among Chinese medical students and to investigate the relationship between these qualities and mental health indicators. Specifically, we seek to answer the following research questions:

1. What are the key dimensions and indicators of professional psychological qualities in Chinese medical students?
2. How reliable and valid is the newly developed scale in assessing these qualities?
3. To what extent do professional psychological qualities influence anxiety and depression levels among medical students, and what is the nature of these relationships?

## 2 Study 1: Development of the medical students’ professional psychological qualities questionnaire

### 2.1 Theoretical model

Strictly speaking, the academic community has yet to reach a unified consensus regarding the structure of professional psychological qualities, and the composition of such qualities varies among different groups. Many scholars have proposed diverse components, including professional values, professional cognition, professional ethics, professional character traits, vocational skills, occupational emotions, occupational adaptability, occupational psychological stress, occupational resilience, occupational communication, occupational efficacy, and professional identity.

The establishment of the three-dimensional theoretical model for professional psychological qualities is supported by a comprehensive review of literature from various fields, including psychology, medical education, and organizational behavior. Research in personality psychology has consistently demonstrated the importance of specific traits, such as conscientiousness, emotional stability, and openness to experience, for job performance and career success across occupations (Barrick and Mount, 1991; Judge et al., 1999). In the medical field, traits like empathy, integrity, and resilience have been identified as crucial for effective patient care and professional well-being (Hojat et al., 2002; Jackson et al., 2007; Tempski et al., 2012). These findings underscore the relevance of including professional psychological traits as a key dimension in the proposed model.

Moreover, the literature on competency-based medical education emphasizes the significance of developing psychological competencies beyond medical knowledge and technical skills (Frank et al., 2010). Studies have highlighted the importance of emotional intelligence, adaptability, communication skills, self-regulation, problem-solving, and interpersonal effectiveness for success in medical practice and career advancement (Kanfer and Ackerman, 1989; Duffy et al., 2004; Stratton et al., 2005; Boyatzis, 2008; Arora et al., 2010). These competencies have been shown to predict job performance, patient satisfaction, and overall career success in the medical profession (Grewal and Davidson, 2008; Lievens and Sackett, 2012). The inclusion of professional psychological competences as a distinct dimension in the model is thus well-supported by empirical evidence.

Furthermore, research on professional identity formation has shown its central role in medical education and its impact on attitudes, behaviors, well-being, job satisfaction, commitment, and performance (Lauver and Kristof-Brown, 2001; Pratt et al., 2006; Monrouxe, 2010; Wilson et al., 2013; Cruess et al., 2015). The development of a strong professional identity has been associated with increased resilience, adaptability, and the ability to cope with the challenges and demands of medical practice (Wald et al., 2015). Additionally, studies have suggested that a well-developed professional identity can foster a sense of meaning, purpose, and alignment with professional values, which contribute to overall job satisfaction and career longevity (Dall'Alba, 2009).

By integrating findings from these diverse domains, the proposed three-dimensional model, encompassing professional psychological traits, competences, and identity, is grounded in a robust theoretical foundation. This interdisciplinary approach offers a comprehensive framework for understanding professional psychological qualities in the context of medical education and practice. The model has the potential to guide future research, inform curriculum design, and support the development of targeted interventions to foster the growth of professional psychological qualities among medical students and practitioners.

### 2.2 Dimension construction

#### 2.2.1 Dimension construction for professional psychological traits and professional psychological competences

This study employed a qualitative research approach, targeting medical professionals as the initial research subjects to explore the professional psychological qualities and abilities required in the medical profession.

The study employed a two-round expert consultation approach, involving the distribution of 35 and 400 questionnaires, respectively. The first round of expert consultation utilized a semi-structured interview questionnaire survey, inviting participation from 3 medical experts with senior professional titles, 2 experts in higher medical education with senior titles, and 30 frontline clinical doctors from 7 provinces and cities, including Beijing, Shanghai, Jiangsu, Guangdong, Sichuan, Henan, and Chongqing. Among the participants, 10 held bachelor’s degrees, 17 held master’s degrees, and 8 held doctoral degrees. Additionally, 10 participants held intermediate professional titles, 10 held associate senior titles, and 15 held senior titles. A total of 35 valid questionnaires were collected, achieving a 100% response rate. The interviews aimed to elicit opinions on personal abilities, individual characteristics, and psychological attributes deemed necessary for effective medical practice. Subsequently, the collected vocabulary was coded, resulting in the identification of 43 terms, including proactivity, empathy, communication skills, and adaptability, which formed the “Precision Survey of Professional Psychological Qualities for Medical Professionals.”

In the second round of expert consultation, the “Precision Survey of Professional Psychological Qualities for Doctors” was administered to 400 medical staff and patients across 6 tertiary hospitals in Chengdu. Participants were asked to rate the importance of each term on a 10-point scale from the provided list of vocabulary. A total of 359 valid questionnaires were collected for statistical analysis, representing an 89.75% response rate. The statistical analysis expert judgment coefficient Ca was 0.6, and the familiarity coefficient with the survey content, Cs, was 0.8, resulting in an expert authority coefficient of 0.7, which met the statistical indicators for the expert consultation method. Furthermore, regarding the degree of consensus among experts based on the statistical analysis, the coefficient of variation (CV) ranged from 0 to 0.32, and Kendall’s *W* value was 0.37 (*p* < 0.001). Generally, a CV value of less than 0.25 and a Kendall’s coefficient between 0.4 and 0.5 are considered optimal. In this study, the relatively large sample size in the second expert consultation may have influenced the fluctuations in the CV value and Kendall’s *W* value.

The average importance rankings (s/f) were calculated for each term representing medical professionals’ professional psychological qualities and abilities. The top 10 ranked terms were selected, and those with a selection frequency of less than 50% were removed. As a result, six professional psychological qualities terms were identified: sense of responsibility, meticulous and scientific attitude, positivity, empathy, patience, and meticulousness, with s/f ranging from 2.84 to 6.13. Additionally, 10 occupational psychological abilities terms were identified: communication, professional ethics, adaptability, assessment and judgment, teamwork, stress resistance, learning, practice, emotional self-regulation, and self-protection, with s/f ranging from 3.30 to 6.70.

Considering the inherent differences between medical students and practicing physicians, the six dimensions representing professional psychological qualities obtained from physicians were all transformed into six dimensions representing medical student professional psychological qualities. Additionally, adjustments were made to the 10 terms representing occupational psychological abilities obtained from physicians, with the removal of three terms: professional ethics, assessment and judgment, and self-protection. It was deemed that professional ethics falls under the category of professional identity, while assessment and judgment, as well as self-protection, are more indicative of the technical proficiency required by medical students rather than occupational psychological abilities. Consequently, the remaining seven terms representing occupational psychological abilities were converted to those applicable to medical students.

Based on this, we initially obtained six secondary dimensions representing medical student professional psychological traits: sense of responsibility, meticulousness, positivity, empathy, patience, and attention to detail. Additionally, we identified seven secondary dimensions representing medical student professional psychological competences: communication skills, adaptability, teamwork skills, resilience, learning abilities, practical skills, and emotional self-regulation. These 13 terms were used as the initial secondary dimensions for questionnaire development.

#### 2.2.2 Dimension construction for professional identity

Building upon existing research on medical student professional identity and drawing from previous studies, the intrinsic structure of medical student professional identity is proposed to include six factors: professional cognition, professional emotion, professional behavior, professional commitment, professional expectations, and professional values. These factors were integrated into six secondary dimensions for the initial questionnaire development in this study.

### 2.3 Questionnaire development

#### 2.3.1 Initial questionnaire preparation

Based on the interview results, preliminary questionnaire survey findings, and the theoretical framework mentioned above, as well as reference to other questionnaires related to medical student qualities and professional psychological traits, an initial questionnaire for “Medical Student Professional Psychological Qualities” was developed. After the initial questionnaire items were compiled, five medical professors, five medical students, and five practicing physicians were invited to conduct a pilot test. The purpose was to identify any ambiguities or unclear expressions in the items, aiming to further refine the wording for clarity and understanding. Ultimately, a preliminary questionnaire consisting of 106 items was formulated. All items were rated on a 5-point Likert scale (ranging from “completely agree” to “completely disagree”), using a single-choice forced response format, with ratings based on the degree of self-alignment by medical students. Additionally, some items were scored in reverse.

The participants of this study were medical students from Chengdu University of Traditional Chinese Medicine. The initial questionnaire was disseminated through an online platform, resulting in the collection of 305 responses. Among these, 300 responses were deemed valid, with 79 male participants and 221 female participants. Data analysis and processing were conducted using SPSS version 25.0. Through item analysis, items with factor loadings below 0.30 (Hair et al., 2019), communalities below 0.20 (Costello and Osborne, 2005), significance levels above 0.05 (Field, 2018), and item-total correlations below 0.20 (Streiner et al., 2015) were removed. A total of 15 items were deleted, resulting in the final version of the “Medical Student Professional Psychological Qualities” questionnaire consisting of 91 items.

#### 2.3.2 Formal questionnaire test

Convenience sampling was employed in this study, selecting students from Chengdu University of Traditional Chinese Medicine, North Sichuan Medical College, and West China School of Medicine at Sichuan University as participants. The formal questionnaire was distributed through an online platform, resulting in the collection of 837 responses. Among these, 800 responses were considered valid, comprising 436 from Chengdu University of Traditional Chinese Medicine, 121 from North Sichuan Medical College, 147 from West China School of Medicine at Sichuan University, and 96 from other medical schools. The gender distribution included 219 male participants and 581 female participants. Additionally, there were 384 participants majoring in Traditional Chinese Medicine, 302 in Western Medicine, and 114 in Integrated Chinese and Western Medicine. Regarding academic year, there were 241 freshmen, 212 sophomores, 235 juniors, and 112 seniors.

The 400 valid questionnaires were randomly divided into two equal groups: one group of 400 was utilized for exploratory factor analysis to initially construct the theoretical model of medical students’ professional psychological quality, while the other 400 were used for confirmatory factor analysis to confirm the structure of the formal questionnaire. Internal consistency of the questionnaire was assessed using Cronbach’s α coefficient. Correlation analysis, exploratory factor analysis, and reliability analysis were conducted using SPSS 25.0, while confirmatory factor analysis was performed using AMOS 26.0.

### 2.4 Testing of validity and reliability

#### 2.4.1 Exploratory factor analysis

The questionnaire consists of three subscales: professional psychological traits, professional psychological competence, and professional identity, each of which underwent exploratory factor analysis (EFA). Results indicated that the professional psychological traits subscale had a Kaiser-Meyer-Olkin (KMO) measure of sampling adequacy of 0.879, and Bartlett’s test of sphericity yielded a significant result (χ^2^ = 3,853.30, *p* < 0.001); the professional psychological competence subscale had a KMO value of 0.865, with Bartlett’s test statistic being 4,451.54 (*p* < 0.001); the professional identity subscale had a KMO value of 0.943, with Bartlett’s test statistic being 8,763.38 (*p* < 0.001). These findings suggest that there were shared factors among the items, supporting the suitability for exploratory factor analysis (Field, 2018; Hair et al., 2019). Principal component analysis with varimax rotation was conducted, and factors were extracted based on eigenvalues greater than 1 (Patil et al., 2017), while considering the scree plot to determine the number of factors to retain (Raîche et al., 2013), with criteria for item removal including communalities less than 0.3, factor loadings below 0.4 (Howard, 2016), and items loading across multiple factors (Carpenter, 2018).

The professional psychological traits subscale yielded five factors, accounting for 60.945% of the total variance; the professional psychological competence subscale yielded four factors, explaining 57.585% of the total variance; and the professional identity subscale yielded four factors, explaining 66.234% of the total variance. Ultimately, this led to the development of the formal questionnaire, titled “Medical Students’ Vocational Psychological Qualities,” comprising three subscales, three primary factors, 13 secondary factors, and 61 items (Table 1).

**Table 1:** Exploratory factor analysis results of each subscale of the questionnaire on Professional psychological Qualities of medical students (*n* = 400).

Subscale	Dimensions	Items	RVC (%)	Eigenvalue
Professional psychological traits	1 Responsibility	7	18.585	7.363
	2 Affinity	5	14.446	2.062
	3 Positivity	4	9.689	1.818
	4 Patience	3	9.451	1.151
	5 Carefulness	3	8.774	1.014
Professional psychological competence	1 Learning in practice	6	15.724	5.532
	2 Teamwork	4	14.876	2.17
	3Psychological resilience	4	14.703	1.469
	4 Communication	4	12.282	1.194
Professional identity	1 Commitment	6	22.931	9.35
	2 Expectation	7	18.755	1.688
	3 Understanding	4	12.785	1.515
	4 Behavior tendency	4	11.763	1.357

Based on the content of each factor item and relevant theoretical research, the factors are named as follows (Table 1): Factor 1 in the professional psychological traits subscale reflects the quality of medical students being thoughtful and goal-oriented, adhering to social norms by thinking before acting, and is named “Responsibility. “Factor 2 reflects the tendency of medical students to engage in close contact with patients, colleagues, etc., and is named “Affability.” Factor 3 represents the proactive and optimistic attitude of medical students in their personal and professional lives, and is named “Positivity.” Factor 4 embodies the trait of medical students being patient when dealing with patients and clinical affairs, and is named “Patience.” Factor 5 involves the conscientious and meticulous participation of medical students in medical activities, and is named “Carefulness.”

In the professional psychological competence subscale, Factor 1 reflects the ability of medical students to master or innovate clinical skills and treatment techniques through learning and practical activities, and is named “Learning in Practice.” Factor 2 involves the ability of medical students to collaborate with team members to achieve goals and succeed together, and is named “Teamwork.” Factor 3 embodies the ability of medical students to adapt to various challenges in work, adopt positive coping strategies, and quickly manage negative emotions, and is named “Psychological Resilience.” Factor 4 reflects the ability of medical students to handle pressure and setbacks calmly, and effectively manage interpersonal relationships through good communication, and is named “Communication.”

In the professional identity subscale, Factor 1 involves medical students’ preferences, loyalty, attachment, and the likelihood of changing careers in the medical profession, as well as their awareness of the costs of leaving the medical profession, and is named “Commitment.” Factor 2 reflects medical students’ expectations of the level of development and success in the medical profession, and is named “Expectations.” Factor 3 embodies medical students’ comprehensive views on the nature, social status, values, and environmental aspects of the medical profession, and is named “Understanding.” Factor 4 reflects medical students’ behavioral tendencies in medical practice, including professional skills and behavioral characteristics, and is named “Behavior tendency.”

#### 2.4.2 Confirmatory factor analysis

**Figure 1:**
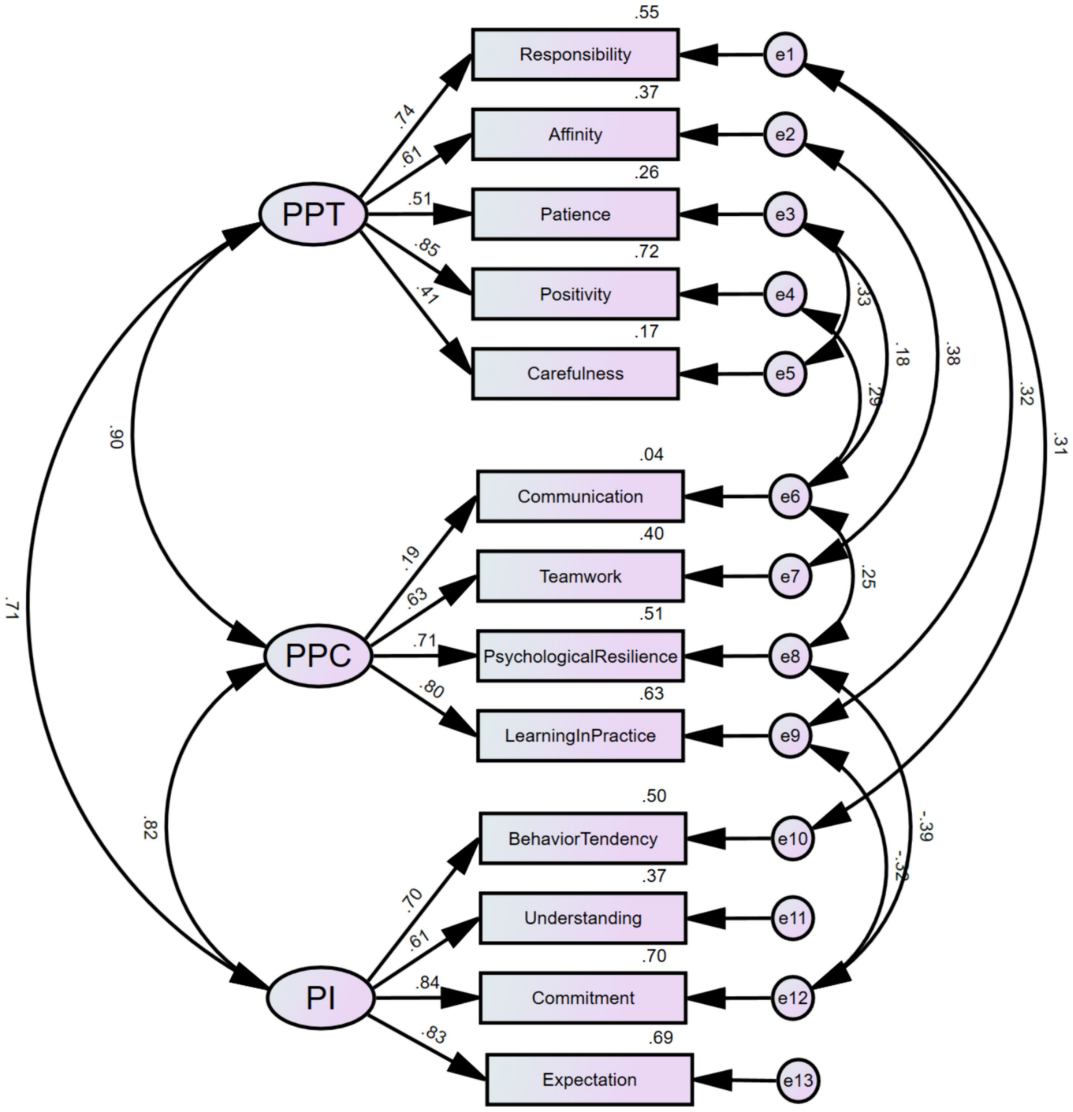
Standardized path coefficient diagram of structural equation model.

Through confirmatory factor analysis, it was found that all fit indices of the theoretical model met the statistical requirements, indicating good construct validity of the questionnaire structure for medical students’ professional psychological qualities (Table 2). The standardized path coefficients of the specific structural variance model are shown in Figure 1.

**Table 2:** Fitting index of confirmatory factor analysis model for each subscale of the questionnaire on Professional Psychological Qualities of Medical students (*n* = 400).

Questionnaires	CMIN/DF	GFI	AGFI	NFI	RFI	IFI	TLI	RMSEA
PPT subscale	2.314	0.909	0.879	0.880	0.854	0.928	0.912	0.057
PPC subscale	1.943	0.936	0.911	0.902	0.878	0.950	0.937	0.049
PI subscale	2.588	0.902	0.870	0.914	0.896	0.945	0.934	0.063
PPQ	2.969	0.945	0.906	0.936	0.906	0.957	0.935	0.070

PPQ, Professional Psychological Qualities; PPC, Professional psychological competence; PI, professional identity; PPT, Professional psychological traits.

Based on the theoretical foundation and suggestions from the modification indices, the model underwent repeated fittings and modifications using maximum likelihood (ML), resulting in the addition of 9 pairs of covariance relationships between residual terms. From a practical standpoint, medical students’ conscientiousness and professional emotions encompass attitudinal components, while their level of professional commitment and conscientious attitude can influence their psychological adaptability and proactivity. Therefore, covariance relationships were added between e1 and e10, e9, as well as e12 and e8, e9. Additionally, an individual’s affability trait can affect their teamwork ability, while patience and positive personal traits can influence communication and coordination skills, which, in turn, affect psychological resilience. Consequently, covariance relationships were included between e2 and e7, e6 and e3, as well as e8 and e4, e6.

#### 2.4.3 Correlation analysis between total questionnaire and subscale

The correlation coefficients (*r*) between the professional psychological traits, professional psychological competence, and professional identity subscales and the total questionnaire ranged from 0.850 to 0.869 (*p* < 0.01). The correlations between the subscales within the professional psychological traits component ranged from 0.666 to 0.820 (*p* < 0.01), within the professional psychological competence component ranged from 0.542 to 0.817 (*p* < 0.01), and within the professional identity component ranged from 0.742 to 0.896 (*p* < 0.01), all showing statistical significance, indicating good internal consistency of the questionnaire.

A multicollinearity diagnosis was performed on the professional personality, professional competence, and professional identity dimensions within the professional psychological qualities structure. The results revealed a variance inflation factor (VIF) of 2.034 for professional traits, 2.280 for professional competence, and 1.590 for professional identity. As all three dimensions have VIF values below 5, it indicates the absence of severe multicollinearity among the subscales.

#### 2.4.4 Content validity

The final questionnaire items underwent thorough scrutiny by medical and psychological experts during the pre-survey phase to ensure the clarity, coherence, and completeness of the expressions, as well as the appropriateness of the latent traits measured by the items. Additionally, medical student representatives were invited to conduct further testing and make corresponding modifications, thereby ensuring the content validity of the questionnaire (see Appendix 1).

#### 2.4.5 Reliability test

The overall questionnaire for medical students’ professional psychological qualities exhibited a Cronbach’s α coefficient of 0.947, while the Cronbach’s α coefficients for the professional psychological traits, professional psychological competences, and professional identity subscales were 0.895, 0.845, and 0.933, respectively. With all internal consistency coefficients exceeding 0.800, both for the total questionnaire and the subscales, it indicates that the reliability of the entire questionnaire meets the requirements of psychometric reliability.

**Figure 2:**
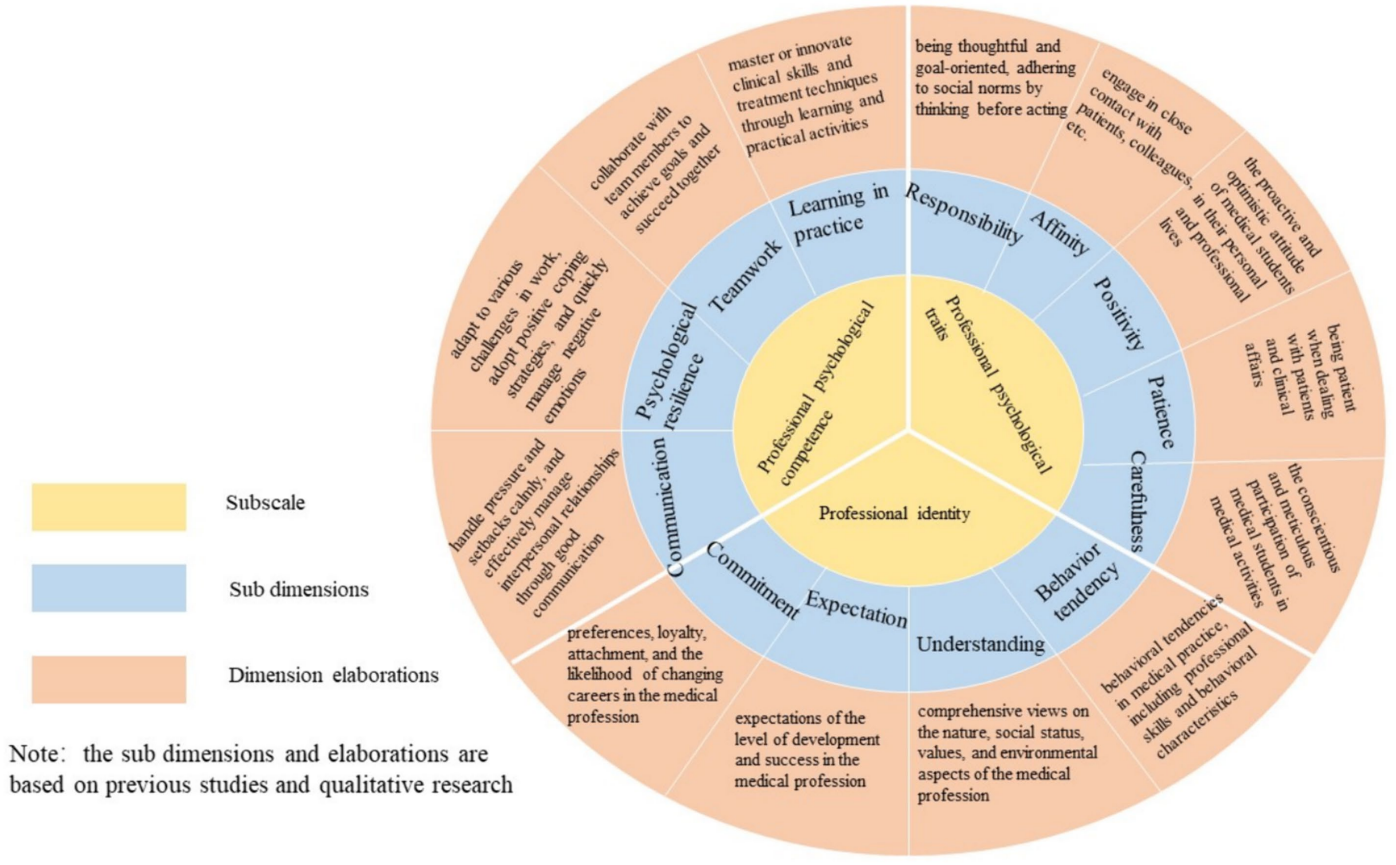
Three-level theoretical structure of professional psychological quality of medical students.

Through the above empirical research, we obtained a three-dimensional theoretical model of professional psychological qualities for medical students (see Figure 2), with the following specific connotations: The professional psychological qualities of medical students inherently encompass professional identity, professional psychological traits, and professional psychological competence. Among these, professional identity is a crucial intrinsic motivational factor that drives medical students to strive for professional competence and persist in the long-term development of their medical career. Professional psychological personality refers to the essential personal characteristics that help medical students adapt to and fulfill the role of a physician. Professional psychological abilities are the critical personal capabilities that enable medical students to adapt to the physician’s role and enhance their clinical competence. These three dimensions interact with and reinforce one another, collectively promoting the development of professional psychological qualities among medical students and facilitating their successful professional development as physicians. Specifically:

1. Professional identity of medical students: This refers to the comprehensive perspective and cognition that medical students hold toward their prospective medical profession. The professional identity of medical students inherently includes four factors: professional cognition, professional expectations, professional commitment, and professional behavioral tendencies.
2. Professional psychological traits of medical students: This refers to the stable psychological characteristics that medical students develop to better fulfill the role of a physician, aligning with the requirements of the medical profession. The professional psychological traits of medical students inherently includes five factors: meticulousness and responsibility, affability and friendliness, positivity and optimism, patience and composure, and attention to detail.
3. Professional psychological competence of medical students: This refers to the psychological coordination competence that medical students develop to better cope with the demands of the physician’s role and behavior, manage professional stress, and engage in self-psychological adjustment. The professional psychological competence of medical students inherently include four factors: learning and practice, teamwork and collaboration, psychological resilience, and communication and coordination.

## 3 Study 2: The relationship between medical students’ professional psychological qualities and mental health

Study 1 utilized a series of qualitative and quantitative studies to develop a medical student professional psychological quality scale that meets the reliability and validity requirements for research purposes. Study 2 will employ this scale to investigate how Professional Psychological Qualities affect anxiety and depression among medical students, delving deeper into their underlying relationships.

Medical students face unique challenges and stressors throughout their education and training, which can lead to an increased risk of developing mental health problems, such as anxiety and depression (Dyrbye et al., 2006; Rotenstein et al., 2016). These mental health issues can have a significant impact on students’ personal and professional lives, making it crucial to identify the psychological qualities that may protect against or mitigate their development. Previous research has shown that certain psychological qualities, such as resilience and emotional regulation, are associated with better mental health outcomes among medical students (Rahimi et al., 2014; Shi et al., 2015). However, the specific mechanisms through which these qualities influence mental health remain unclear. Study 2 aims to address this gap by examining the mediating role of anxiety in the relationship between professional psychological qualities and depression among medical students. By gaining a deeper understanding of these relationships, we can inform the development of targeted interventions and support systems that promote the mental health and well-being of medical students.

The theoretical basis for Study 2 draws upon two key frameworks: the transdiagnostic approach to understanding mental health disorders (Norton and Paulus, 2017) and the tripartite model of anxiety and depression (Clark and Watson, 1991). The transdiagnostic approach highlights the importance of identifying shared vulnerabilities and protective factors that can influence the occurrence of various mental health disorders, including anxiety and depression. This perspective suggests that there are common underlying mechanisms that contribute to the development and maintenance of these disorders. On the other hand, the tripartite model posits that while anxiety and depression share a common component of negative affect, they also have distinct features. Anxiety is characterized by physiological hyperarousal and worry, whereas depression is marked by low positive affect and anhedonia. This model provides a framework for understanding the unique and overlapping aspects of anxiety and depression, and how they may be differentially influenced by psychological factors. In the context of Study 2, these theoretical perspectives inform the hypothesis that professional psychological qualities may serve as protective factors against the development of anxiety and depression among medical students. Furthermore, the tripartite model supports the examination of anxiety as a potential mediator in the relationship between these qualities and depression, as anxiety may be a more proximal outcome that subsequently influences the occurrence of depressive symptoms.

Numerous studies have investigated the prevalence of anxiety and depression among medical students, consistently demonstrating that these mental health problems are more common in this population compared to the general public and age-matched peers (Dyrbye et al., 2006; Rotenstein et al., 2016). In addition to establishing the high prevalence of these issues, researchers have also explored the role of various psychological factors in influencing the mental health of medical students. For example, Rahimi et al. (2014) found that higher levels of resilience were associated with lower levels of anxiety and depression among Iranian medical students. Similarly, Shi et al. (2015) reported that resilience mediated the relationship between stress and depressive symptoms in Chinese medical students, with anxiety serving as a potential underlying mechanism. These findings suggest that resilience may be a key protective factor against the development of anxiety and depression in this population. Other psychological qualities, such as emotional intelligence and coping strategies, have also been linked to mental health outcomes in medical students. Gupta et al. (2015) found that higher emotional intelligence was associated with lower levels of anxiety and depression among medical students in India, while a systematic review by Fares et al. (2016) highlighted the importance of adaptive coping strategies, such as problem-focused coping and seeking social support, in mitigating the negative impact of stress on medical students’ mental health.

Despite these valuable insights, there is a need for further research that specifically examines the mediating role of anxiety in the relationship between professional psychological qualities and depression. Study 2 aims to address this gap by investigating the interplay between these variables and contributing to a more comprehensive understanding of the factors that influence the mental health and well-being of medical students.

Based on the previous analysis, we raised the following three hypotheses:

*Hypothesis 1:* Professional psychological qualities, such as resilience and emotional regulation, are negatively associated with both anxiety and depression among medical students. This hypothesis is based on the transdiagnostic approach, which suggests that these qualities may serve as protective factors against the development of both anxiety and depression.

*Hypothesis 2:* Anxiety is positively associated with depression among medical students. This hypothesis draws upon the tripartite model of anxiety and depression (Clark and Watson, 1991), which suggests that while anxiety and depression share a common component of negative affect, anxiety is characterized by physiological hyperarousal and worry, which may contribute to the development of depressive symptoms.

*Hypothesis 3:* Anxiety mediates the relationship between professional psychological qualities and depression among medical students. This hypothesis builds upon the rationale for Hypotheses 1 and 2 and is informed by the transdiagnostic approach, which highlights the importance of identifying shared vulnerabilities and protective factors that influence the occurrence of mental health disorders. In this case, anxiety potentially serves as a mediating mechanism through which professional psychological qualities influence the development of depressive symptoms in medical students.

These hypotheses aim to explore the complex relationships between professional psychological qualities, anxiety, and depression among medical students, ultimately contributing to a more nuanced understanding of the factors that influence their mental health and well-being. By testing these hypotheses, Study 2 can inform the development of targeted interventions and support systems to promote the mental health of medical students.

### 3.1 Research tool

#### 3.1.1 SAS

The Self-Rating Anxiety Scale (SAS), developed by Zung in 1971, is a widely utilized tool for measuring anxiety levels. It consists of 20 items covering various anxiety symptoms like nervousness and tension, with respondents rating the frequency or severity of each symptom over a specified period. The SAS yields a total score indicative of an individual’s overall anxiety level, with higher scores indicating greater anxiety severity. Despite its brevity and ease of administration, the SAS may not capture all aspects of anxiety and is often supplemented with other measures for a comprehensive assessment. Nevertheless, its versatility, reliability, and extensive validation across diverse populations make it valuable for assessing anxiety in various cultural contexts.

#### 3.1.2 SDS

The Self-Rating Depression Scale (SDS), developed by William W.K. Zung in 1965, is a widely utilized tool for evaluating depressive symptoms in individuals. Comprising 20 items that assess affective, psychological, and somatic symptoms associated with depression, such as sadness and fatigue, respondents rate the frequency or severity of each symptom over a specified period. The total score provides an overall measure of depressive symptomatology, with higher scores indicating greater depression severity. Widely employed in clinical and research settings for screening, treatment assessment, and symptom monitoring, the SDS is valued for its brevity, ease of use, and reliability. Despite its strengths, the SDS, like all self-report measures, may be subject to response biases and might not capture all aspects of depression. Therefore, it is often combined with other measures for a comprehensive assessment of depressive symptoms.

### 3.2 Research procedure

This study employed a convenience sampling method to select students from several medical-related institutions, including Chengdu University of Traditional Chinese Medicine, North Sichuan Medical College, and West China School of Medicine at Sichuan University, as the study participants. A total of 1,000 questionnaires were distributed via online platforms. During the preliminary data screening process, samples with missing key demographic variables or incomplete responses were excluded. Ultimately, 972 valid questionnaires were obtained, resulting in a response rate of 97.2%.

### 3.3 Analysis and result

#### 3.3.1 Demographic characteristics of the subjects

The descriptive statistics from Table 3 outline the demographic profile of 972 respondents. According to the latest published “*2022 Chinese Health Statistics Yearbook*,” the proportion of females among healthcare professionals in China was 72.4% in 2020 and 73% in 2021. In our study, the percentage of female participants (70.37%) closely matches the gender distribution in the healthcare workforce, which aids in the generalizability and applicability of our research findings. For healthcare settings with a more balanced gender ratio, our conclusions require further validation. Most respondents are aged 20–29 (93.42%) and hails mostly from villages (45.88%). Academic qualifications are primarily at the master’s level (87.65%), with 12.35% holding PhDs. Clinical experience ranges from 1 to 4 years, with the majority having 1 or 2 years (54.12 and 38.07%, respectively). In terms of academic grade, the largest proportion are in the first year of their master’s program (55.14%).

**Table 3:** Descriptive statistics for sample 2 (*N* = 972).

variables	category	Frequency	Percent(%)
Gender	Male	288	29.63
	Female	684	70.37
Origin	City	335	34.47
	Town	191	19.65
	Village	446	45.88
Age	20–29	908	93.42
	30–39	60	6.17
	40 above	4	0.41
Degree	Master	852	87.65
	PhD	120	12.35
Clinical years	1 year	526	54.12
	2 year	370	38.07
	3 year	39	4.01
	4 year	37	3.81
Grade	Master 1	536	55.14
	Master 2	190	19.55
	Master 3	126	12.96
	PhD 1	79	8.13
	PhD 2	30	3.09
	PhD 3	11	1.13

#### 3.3.2 Variable correlation matrix

Table 4 displays the correlation coefficients between the main variables in the study. The Anxiety score shows significant positive correlations with the Depression score (*r* = 0.316, *p* < 0.01), Professional Psychological qualities(PPQ) (*r* = 0.352, *p* < 0.01), Professional Psychological competence (PPC) (*r* = 0.304, *p* < 0.01), and Professional Identity (PI) (*r* = 0.344, *p* < 0.01). Additionally, the Depression score exhibits positive correlations with PPC (*r* = 0.022, *p* > 0.05), PPC (*r* = 0.116, *p* < 0.01), and PI (*r* = 0.005, *p* > 0.05), although only the correlation with PPC reaches statistical significance. PPC demonstrates a strong positive correlation with PPC (*r* = 0.907, *p* < 0.01) and PI (*r* = 0.938, *p* < 0.01), indicating high interrelatedness among these variables. Similarly, PPC and PI exhibit a robust positive correlation (*r* = 0.769, *p* < 0.01). Moreover, the variable Professional Psychological traits (PPT) displays positive correlations with all other variables, though only the correlation with PPQ reaches statistical significance (*r* = 0.321, *p* < 0.01). These findings underscore the interconnectedness of Professional Psychological qualities, mental abilities, and identity among the study participants, highlighting the complex nature of these constructs within the context of the research domain.

**Table 4:** Correlations between main variables.

	Anxiety score	Depression score	PPQ	PPC	PI
Depression score	0.316^**^	1			
PPQ	0.352^**^	0.022	1		
PPC	0.304^**^	0.116^**^	0.907^**^	1	
PI	0.344^**^	0.005	0.938^**^	0.769^**^	1
PPT	0.321^**^	−0.035	0.928^**^	0.804^**^	0.782^**^

PPQ, professional psychological qualities; PPC, professional psychological competence; PI, professional identity; PPT, professional psychological traits.

*P < 0.05, **P < 0.01, ***P < 0.001.

#### 3.3.3 Simple mediation analysis

In mediation analysis, a masking effect occurs when the indirect (mediated) effect of an independent variable on a dependent variable through a mediator variable is stronger or more significant than the direct effect of the independent variable on the dependent variable. In Table 5, we observe a masking effect in the relationship between Professional Psychological qualities(PPQ) and Depression score. Specifically, the direct effect of PPQ on Depression score is estimated to be −0.0232, but it is not statistically significant as the 95% confidence interval (−0.0377 to −0.0086) includes zero.

**Table 5:** Bootstrap analysis of mediated effect significance test.

	Effect value	BootSE	BootLLCI	BootULCI
Direct effect (PPQ-SDS)	−0.0232	0.0074	−0.0377	−0.0086
Mediated effects (PPQ-SAS-SDS)	0.0283	0.0052	0.0187	0.0390
Total effect (PPQ-SDS)	0.0051	0.0074	−0.0093	0.0196

However, when considering the mediated effect of PPQ on Depression score through the mediator variable Anxiety score, we find a statistically significant mediated effect (0.0283) with a confidence interval (0.0187 to 0.0390) that does not include zero. This indicates that PPQ influences Depression score indirectly through Anxiety score. The presence of a significant mediated effect but a non-significant direct effect suggests that the relationship between PPQ and Depression score is primarily driven by the mediating variable Anxiety score. In other words, the effect of PPQ on Depression score is fully explained by its influence on Anxiety score, suggesting that Anxiety score mediates the relationship between PPC and Depression score. This phenomenon is characteristic of a masking effect, where the mediated pathway obscures the direct relationship between the independent and dependent variables.

**Figure 3:**
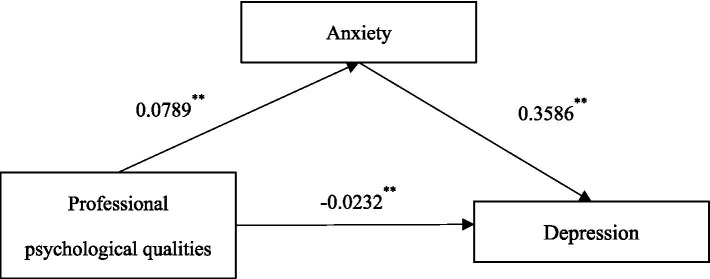
Simple mediation effect diagram. *p < 0.05, **p < 0.01, ***p < 0.001.

In this case, the simple mediation effect diagram (see Figure 3) provides an intuitive representation of the mediated effect found in Table 5. The diagram illustrates the relationship pathways between the independent variable (PPQ), the mediator variable (Anxiety score), and the dependent variable (Depression score). From the diagram, it is evident that the independent variable PPQ influences the dependent variable Depression score both directly and indirectly (through the mediator variable Anxiety score). This indirect pathway contributes to the total effect of the independent variable PPQ on the dependent variable Depression score. Since the positive and negative coefficients of direct effect and indirect effect are opposite, the two cancel each other, resulting in the intermediary mechanism that makes the result with insignificant overall effect appear. So called a masking effect occurs, indicating the crucial mediating role of the mediator variable Anxiety score between the independent variable PPQ and the dependent variable Depression score. The visualization of the simple mediation effect diagram helps in intuitively understanding and explaining the relationships between variables in the mediation model, providing crucial support for the visual presentation of research findings. In summary, Hypothesis 1 is partially supported, with evidence for the negative association between professional psychological qualities and depression but a positive association with anxiety. Hypothesis 2 is supported, confirming the positive association between anxiety and depression among medical students. Hypothesis 3 is supported, highlighting the crucial mediating role of anxiety in the relationship between professional psychological qualities and depression among medical students.

## 4 Discussion

Overall, to explore the professional psychological qualities of Chinese medical students, we conducted two studies. We developed a theoretical model, created a standardized psychological measurement tool, and examined its relationship with anxiety and depression. Study 1 involved extensive qualitative and quantitative research, resulting in a three-tiered structure of professional psychological qualities. After multiple rounds of calibration and validation, we obtained a psychometric scale with satisfactory reliability and validity for the purposes of our research. Building upon Study 1, Study 2 utilized the standardized professional psychological qualities scale to investigate the relationship between these qualities and anxiety and depression among a sample of 942 participants. Our findings revealed a masking effect of anxiety as a mediator in the impact of professional psychological qualities on depression. In our discussion of the research results, we will focus on three aspects: the development of research tools, the relationship between professional psychological qualities and mental well-being, and the practical applications of our findings.

### 4.1 Questionnaire development

Prior research has evidenced a concerning decline in student psychological well-being, paralleled by a steady rise in school psychological crisis interventions. Consequently, the imperative to enhance students’ psychological resilience cannot be overstated. As such, the development of a theoretical framework elucidating the professional psychological qualities of medical students stands as foundational within the realm of psychological resilience education research. By delineating the constituent elements of medical students’ professional psychological qualities, educators are empowered to tailor cultivation programs to address specific deficiencies in students’ psychological attributes, thereby adopting a personalized approach to education. The formulation of a questionnaire targeting medical students’ professional psychological qualities serves a dual purpose: firstly, to gain insights into the current landscape of medical students’ professional psychological attributes, facilitating targeted interventions to address gaps in psychological resilience within educational curricula; secondly, to serve as a tool for identifying and selecting individuals possessing robust professional psychological qualities, thus contributing to the cultivation of a cadre of professionals adept in navigating the demands of the medical profession.

The cultivation of professional psychological traits and capabilities among medical students is fundamentally aimed at enhancing their ability to adapt to and serve effectively in clinical practice settings. Recognizing this imperative, our study employed an empirical research approach to investigate the expectations of both medical professionals and patients regarding the professional psychological traits and capabilities required of physicians. This empirical inquiry served as the foundation for delineating the specific attributes comprising professional psychological traits and capabilities. The theoretical framework of the professional psychological qualities of medical students, thus developed, embodies a dual-layered structure encompassing three primary factors and 13 secondary factors. By integrating empirical research findings with theoretical insights, our study not only offers practical implications but also advances innovative perspectives within the field of professional psychological trait education.

The meticulous development of the questionnaire adhered rigorously to established protocols for questionnaire construction, with a comprehensive review by expert evaluators ensuring the content validity of the instrument. The confirmatory factor analysis further attested to the robustness of the questionnaire’s structural model, demonstrating a favorable fit between the proposed factors. Notably, the questionnaire exhibited significant and moderately high correlations across its factors, indicating substantive relationships among the measured constructs. Moreover, the achieved internal consistency reliability met the requisite standards for group administration, bolstering the questionnaire’s reliability in assessing the professional psychological qualities of medical students. Importantly, the conceptual framework underpinning the questionnaire design drew from both extant research literature and theoretical foundations, bolstering its validity and relevance. This comprehensive validation process underscores the questionnaire’s adherence to the principles of psychometric measurement, rendering it suitable for assessing the professional psychological traits of medical students. Consequently, it holds promise for facilitating research endeavors in the domain of medical students’ professional psychological traits and their implications for psychological well-being. And its utility extends to informing the development of tailored educational interventions aimed at nurturing the professional development of medical students.

### 4.2 Professional psychological qualities and mental health

Based on our research findings, the total effect of professional psychological qualities on depression among medical students was not significant. However, the results of the mediation analysis revealed that professional psychological qualities had a negative direct effect on depression, which is consistent with previous studies. What sets our findings apart is that we discovered a positive relationship between professional psychological qualities and anxiety levels among medical students. This increased anxiety, in turn, contributed to higher levels of depression, thus creating a positive indirect effect of professional psychological qualities on depression.

The direct effect of professional psychological qualities on depression among medical students was found to be statistically insignificant. However, through a mediation analysis, it was revealed that professional psychological qualities had a negative indirect effect on depression via the mediating factor of anxiety. This suggests that while the direct impact of professional psychological qualities on depression may not be significant, it can still influence depression levels indirectly through its association with anxiety. This highlights the intricate interplay between psychological factors like anxiety and depression among medical students (Liu et al., 2021, 2023; Geng et al., 2022; Zhang et al., 2022).

The study findings suggest that higher levels of professional psychological qualities were associated with increased anxiety among medical students (Dyrbye et al., 2006; Hope and Henderson, 2014; Chen et al., 2022). Additionally, elevated anxiety levels were linked to higher levels of depression among these medical students, acting as a mediator between professional psychological qualities and depression, resulting in a positive indirect effect (Pelaccia et al., 2021; Iftikhar et al., 2022). These results highlight the intricate relationship between psychological factors like anxiety and depression in the context of medical education, emphasizing the necessity for targeted interventions to support the mental well-being of medical students.

The coping strategies utilized by medical students are pivotal in influencing their experience of anxiety and depression. Factors such as self-awareness, professional identity, social support, and psychological coping abilities can significantly impact how medical students manage stress and emotional challenges (Park and Adler, 2003; Wu et al., 2020; Li et al., 2021). Anxiety tends to be associated with negative coping mechanisms and a lack of effective stress management skills, while depression may stem from self-doubt, uncertainty in professional identity, and strained interpersonal relationships (Rummell, 2015; Ramadianto et al., 2022). Positive self-awareness and a strong professional identity have been shown to mitigate anxiety symptoms, whereas inadequate coping skills and decreased adaptability in dealing with professional challenges may exacerbate depression (Brower, 2021).

Understanding these mechanisms is crucial for implementing targeted psychological interventions and support measures to enhance medical students’ professional psychological quality and overall well-being (Huang et al., 2021; Zhao et al., 2021). By recognizing the interplay between coping strategies, mental health outcomes, and external factors such as social support and resilience, educational institutions and healthcare providers can tailor interventions to better support the mental health needs of medical students. Encouraging adaptive coping strategies, promoting self-awareness, and fostering a supportive environment can contribute to the well-being and resilience of medical students, ultimately enhancing their ability to navigate the challenges of medical education and practice (Pan and Hao, 2022; Dai et al., 2023).

The unique contribution of our study lies in uncovering this indirect relationship and shedding light on how professional psychological qualities can influence depression levels among medical students through the mediating factor of anxiety. These findings have significant implications for understanding the complex dynamics between professional psychological qualities, anxiety, and depression among medical students. They emphasize the importance of considering the role of anxiety in the relationship between professional psychological qualities and depression. Addressing anxiety levels may be crucial in mitigating the potential negative impact of professional psychological qualities on depression among medical students.

### 4.3 Practical implications

The findings of our study hold significant practical implications for the training and development of future physicians. The identified primary and secondary factors can effectively inform curriculum development, guiding the design of learning objectives and content related to professional psychological traits and capabilities. Integrating these factors into medical education programs ensures comprehensive training in these essential qualities. The specific attributes identified in the study can guide the development of skill-building activities and interventions aimed at fostering these traits and capabilities among medical students. Mentorship programs can leverage experienced physicians as role models to impart and demonstrate desired qualities, providing guidance and feedback throughout students’ training. Additionally, the identified factors can inform the development of valid assessment tools and methods to evaluate students’ acquisition and demonstration of these traits. Continuing professional development programs for practicing physicians can also benefit from integrating these findings to enhance clinical effectiveness and patient care. However, it’s crucial to tailor the practical application of these findings to the specific context and needs of each medical education program, collaborating with educators, administrators, and stakeholders to ensure effective implementation and integration into curricula. This approach promotes the development of well-rounded and competent physicians.

The masking effect uncovered in our study has significant implications for comprehending and tackling the mental health requirements of medical students. The discovery that professional psychological qualities have a negative direct impact on depression but a positive indirect influence through anxiety underscores the intricate interplay between these factors. It implies that interventions focused solely on enhancing professional psychological qualities may not suffice to alleviate depression among medical students. Instead, a more holistic approach that simultaneously addresses both professional psychological qualities and anxiety levels is essential. This could entail integrating stress management techniques, coping strategies, and mental health support services into medical education curricula. By targeting both the direct and indirect pathways through which professional psychological qualities affect depression, we can develop more efficacious interventions to foster the well-being of medical students. Moreover, the masking effect highlights the necessity for continuous monitoring and assessment of mental health outcomes among medical students, as the relationship between professional psychological qualities and depression may be obscured by the mediating role of anxiety. Regular screening for anxiety and depression, coupled with targeted support for students identified as being at risk, can help ensure that the mental health needs of medical students are adequately addressed.

### 4.4 Limitations and prospect

The study provides valuable insights into the relationship between professional psychological qualities and mental health outcomes among medical students, yet several limitations warrant consideration. Firstly, the reliance on self-report measures introduces the potential for biases, such as social desirability or response bias, which may affect the accuracy and reliability of the results. As the construction of the theoretical model primarily relies on localized research in China, although it has been well-validated for reliability and validity within the Chinese medical student population, its robustness across different sociocultural backgrounds warrants further cross-cultural validation. Additionally, the cross-sectional design precludes the establishment of causal relationships between professional psychological traits and mental health outcomes. Thus, longitudinal studies are needed to delineate the temporal dynamics and causal pathways more comprehensively. At last, the study’s sample primarily comprised students from specific institutions, limiting the generalizability of the findings. In the scale development process, we placed emphasis on establishing discriminant validity between the dimensions of professional psychological qualities. While we conducted factor analysis and evaluated the correlations and collinearity among dimensions, further refinement of the scale items or dimensionality may be warranted to ensure optimal discriminant validity. Future research endeavors should continue to validate and refine the scale to enhance its psychometric robustness.

Future research should aim to replicate these results in more diverse samples to enhance their external validity. While the study explored mediation effects, it did not investigate other potential moderators or mediators that may influence these relationships, such as social support or coping strategies. Future research could address these gaps and also examine the effectiveness of interventions targeting professional psychological traits in promoting the psychological well-being and professional development of medical students longitudinally. While the study contributes to our understanding of the interplay between professional psychological traits and mental health outcomes among medical students, addressing these limitations and conducting further research in these areas will advance our knowledge and inform the development of interventions to support the mental health of future physicians.

## 5 Conclusion

In conclusion, this research aimed to develop a comprehensive tool for assessing the professional psychological qualities of medical students and to investigate the relationships between these qualities, anxiety, and depression. Study 1 successfully constructed a robust psychological measurement tool through a combination of qualitative and quantitative methods. The resulting scale consists of three main components: professional psychological competence, professional psychological traits, and professional identity. These components are further divided into 13 sub-dimensions, which are measured by 61 items. The scale demonstrated satisfactory reliability and validity, making it suitable for the intended research purpose and providing a valuable tool for future studies in this field.

Study 2 applied the newly developed professional psychological qualities scale to evaluate the levels of depression and anxiety among a sample of 972 medical students. The findings from the simple mediation analysis revealed a complex relationship between professional psychological qualities, anxiety, and depression. Notably, professional psychological qualities were found to have a direct negative impact on medical students’ levels of depression, suggesting that cultivating these qualities may help protect against depressive symptoms. However, the analysis also uncovered an indirect positive influence of professional psychological qualities on anxiety levels, which in turn positively affected depression levels. This indirect effect resulted in an overall masking effect, indicating that the relationship between professional psychological qualities and depression levels may be more nuanced than initially anticipated.

The results of this research have important implications for medical education and student well-being. The development of a reliable and valid scale for assessing professional psychological qualities provides a valuable tool for identifying areas of strength and weakness among medical students. This information can be used to design targeted interventions and support programs that foster the development of these essential qualities. Moreover, the findings from Study 2 underscore the need for a comprehensive approach to addressing mental health concerns among medical students. While promoting professional psychological qualities may have a direct positive impact on reducing depressive symptoms, the complex interplay with anxiety highlights the importance of considering multiple factors when developing prevention and intervention strategies.

Future research should build upon these findings by exploring the specific mechanisms through which professional psychological qualities influence anxiety and depression levels. Additionally, longitudinal studies could provide valuable insights into how these relationships evolve over time and how they may be affected by different stages of medical education and training. Finally, the effectiveness of interventions designed to enhance professional psychological qualities and mitigate anxiety and depression among medical students should be rigorously evaluated to inform best practices in medical education and student support services.

## Data availability statement

The raw data supporting the conclusions of this article will be made available by the authors, without undue reservation.

## Ethics statement

The studies involving humans were approved by Academic Ethics Committee of Sichuan Psychological Society. The studies were conducted in accordance with the local legislation and institutional requirements. The participants provided their written informed consent to participate in this study.

## Author contributions

WL: Conceptualization, Formal analysis, Funding acquisition, Investigation, Methodology, Writing – original draft, Writing – review & editing. WF: Conceptualization, Formal analysis, Investigation, Methodology, Software, Visualization, Writing – original draft, Writing – review & editing. YX: Data curation, Investigation, Project administration, Visualization, Writing – original draft, Writing – review & editing. YD: Conceptualization, Data curation, Formal Analysis, Investigation, Project administration, Visualization, Writing – original draft, Writing – review & editing. JD: Conceptualization, Funding acquisition, Investigation, Methodology, Project administration, Supervision, Visualization, Writing – original draft, Writing – review & editing.
