# Supplementary material for: Professional psychological qualities of Chinese medical students: theoretical models, questionnaire development, and relationship with mental health
Source: Front Psychol. 2024 Jul 4;15:1411085. doi: 10.3389/fpsyg.2024.1411085 (PMC11258856; doi:10.3389/fpsyg.2024.1411085)
Supplement: Supplementary file 1 [file Data_Sheet_1.docx]

## Appendix 1

**Questionnaire of Professional Psychological Quality for Medical Students (English)**

| Serial Number | Item | Completely agree | Comparatively agree | Not sure | Comparatively not agree | Completely not agree |
| --- | --- | --- | --- | --- | --- | --- |
|  | I am a person who advocates science. |  |  |  |  |  |
|  | I am a person with a strong sense of responsibility. |  |  |  |  |  |
|  | I believe that as a doctor, one must hold a rigorous and scientific attitude. |  |  |  |  |  |
|  | In my usual study and work, I have always been very clear about my responsibilities. |  |  |  |  |  |
|  | I can always keenly perceive the state of the people around me. |  |  |  |  |  |
|  | I will strive to complete what belongs to my area of responsibility. |  |  |  |  |  |
|  | For the study of medical majors, I like to inquire into the details. |  |  |  |  |  |
|  | People around me often praise me for being affable. |  |  |  |  |  |
|  | I am a person who is easy to get close to others. |  |  |  |  |  |
|  | People who see me for the first time will find me amiable. |  |  |  |  |  |
|  | I can easily get along with the people around me. |  |  |  |  |  |
|  | I am usually enthusiastic and friendly to people. |  |  |  |  |  |
|  | I have an optimistic expectation for my future. |  |  |  |  |  |
|  | I am a positive and sunny person. |  |  |  |  |  |
|  | I believe that there are always more solutions than difficulties. |  |  |  |  |  |
|  | When faced with difficulties, I always choose to face them positively. |  |  |  |  |  |
|  | I always lack patience in doing things. |  |  |  |  |  |
|  | I always do things with patience. |  |  |  |  |  |
|  | I can always listen to others patiently. |  |  |  |  |  |
|  | I like to consider many details when doing things. |  |  |  |  |  |
|  | I am a person who cares about details. |  |  |  |  |  |
|  | I always do things very carefully. |  |  |  |  |  |
|  | I like to learn new things actively. |  |  |  |  |  |
|  | I like self-directed learning. |  |  |  |  |  |
|  | I like to solve practical problems. |  |  |  |  |  |
|  | I am good at applying the theories I have learned to practical problem-solving. |  |  |  |  |  |
|  | I like to do things that require hands-on operation. |  |  |  |  |  |
|  | I like the work mode of solving problems while doing things. |  |  |  |  |  |
|  | I like to work with others. |  |  |  |  |  |
|  | Working with others makes me feel very pleasant. |  |  |  |  |  |
|  | I am good at collaborating with others to complete tasks. |  |  |  |  |  |
|  | I think teamwork is very important to do a good job. |  |  |  |  |  |
|  | I'm used to thinking positively when encountering setbacks. |  |  |  |  |  |
|  | I'm not afraid to face unexpected situations. |  |  |  |  |  |
|  | Whenever I encounter something unhappy, I can adjust quickly. |  |  |  |  |  |
|  | I can deal with unexpected situations calmly. |  |  |  |  |  |
|  | I always worry that I might say the wrong thing. |  |  |  |  |  |
|  | I often don't know how to communicate with others. |  |  |  |  |  |
|  | Whenever I feel stressed, I will feel anxious. |  |  |  |  |  |
|  | My brain goes blank in a stressful situation. |  |  |  |  |  |
|  | The prospect of the medical profession is not good, and I have the desire to change careers. |  |  |  |  |  |
|  | After graduation, I will choose to be a doctor. |  |  |  |  |  |
|  | Being a doctor will be my lifelong career. |  |  |  |  |  |
|  | Medical education has made me more determined to become a doctor. |  |  |  |  |  |
|  | If I could choose again, I would still be a doctor. |  |  |  |  |  |
|  | Being a doctor is my ideal career choice. |  |  |  |  |  |
|  | I have excellent academic performance. |  |  |  |  |  |
|  | I have a solid professional study. |  |  |  |  |  |
|  | I think I have the talent to be a doctor. |  |  |  |  |  |
|  | I have a good grasp of operational skills. |  |  |  |  |  |
|  | I think I am capable of doing the future doctor's work. |  |  |  |  |  |
|  | I have the confidence to become an excellent doctor. |  |  |  |  |  |
|  | I am full of confidence in future employment. |  |  |  |  |  |
|  | Doctors have a relatively high social status. |  |  |  |  |  |
|  | A doctor's efforts and rewards are matched. |  |  |  |  |  |
|  | The medical profession is one of the best professions in the current society. |  |  |  |  |  |
|  | Being a doctor is very respectable. |  |  |  |  |  |
|  | When others say something bad about doctors, I will feel uncomfortable. |  |  |  |  |  |
|  | I will pay attention to the news reports about doctors in the society. |  |  |  |  |  |
|  | I am willing to participate in training lectures and other activities that are helpful for my medical practice. |  |  |  |  |  |
|  | In my interactions with students from other majors, I am proud to be a medical student. |  |  |  |  |  |
